# Immuno-chromatic probe based lateral flow assay for point-of-care detection of Japanese encephalitis virus NS1 protein biomarker in clinical samples using a smartphone-based approach

**DOI:** 10.1039/d2na00463a

**Published:** 2022-08-10

**Authors:** Akanksha Roberts, Drishya Prakashan, Himani Dhanze, Ravi Kumar Gandham, Sonu Gandhi, G. Taru Sharma

**Affiliations:** DBT-National Institute of Animal Biotechnology (NIAB) Hyderabad-500032 Telangana India gandhi@niab.org.in +91-040-23120127; DBT-Regional Centre for Biotechnology (RCB) Faridabad-121001 Haryana India; ICAR-Indian Veterinary Research Institute (IVRI) Bareilly-243122 Uttar Pradesh India

## Abstract

Lateral flow assays (LFAs) are one of the most economical, point-of-care (PoC) diagnostic assays that exploit the colorimetric properties of gold nanoparticles (AuNPs). Up to the best of our knowledge, no rapid antigen-based LFA exists for Japanese Encephalitis Virus (JEV) detection. Herein, we have reported a novel portable sandwich-type LFA for on-site detection of the non-structural 1 (NS1) secretory protein of JEV. In-house JEV NS1 antibodies (Abs) were generated and labelled with AuNPs as immunoprobes. A glass fibre membrane conjugate pad was soaked with AuNPs–Ab solution, while the JEV NS1 Ab and anti-rabbit IgG 2° Ab were coated as the test and control lines, respectively, on a nitrocellulose (NC) membrane. Different layers of the LFA were fabricated and various parameters were standardised for optimum colour intensity development. JEV negative serum samples spiked with JEV NS1 Ags (linear range – 1 pg ml^−1^ to 1 μg ml^−1^) were applied onto the sample pad and the intensity of the red colour developed on the test line increased with increasing concentration of Ag. The visual limit of detection (LOD) determined from the LFA was 10 pg ml^−1^, which corresponded to the LOD determined by the graphical data obtained from ImageJ software and the Colorimeter smartphone application. Furthermore, the colorimetric based immunosensor showed minimal non-specific detection of other closely related flaviviral NS1 Ags in the spiked serum, provided a rapid result within 10 min, showed storage stability up to a month at 4 °C, successfully detected the JEV NS1 protein in clinically infected pig serum samples, and hence, may be developed into a PoC screening diagnostic kit for JEV.

## Abstract 2

Lateral flow assay for rapid detection of the JEV NS1 protein biomarker (NS1) in serum samples incorporating a smartphone-based colorimeter application.

## Graphical abstract

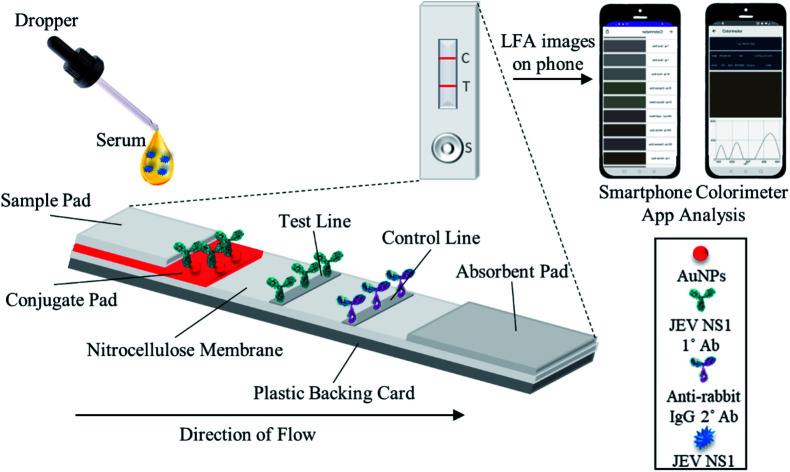

## 1. Introduction

Japanese Encephalitis Virus (JEV) (family Flaviviridae and genus *Flavivirus*)^1^ is the major reason for the spread of mosquito-borne encephalitis in Western Pacific and South-East Asian countries, where it exists as a zoonotic infectious disease. JEV consists of three structural and seven non-structural proteins,^2,3^ out of which the serum secreted non-structural 1 (NS1) protein known to evoke an immunological reaction^4–7^ has been reported as a prospective biomarker for early diagnosis of flaviviral diseases in serum.^8^

Since there is no fool-proof vaccine available against JEV, only palliative therapy can be provided, and the conventional diagnostic techniques such as whole virus isolation, plaque reduction neutralisation test, reverse transcriptase polymerase chain reaction, and enzyme-linked immunosorbent assays (ELISAs)^9^ are time-consuming and laborious, portable diagnostic techniques resulting in rapid detection of trace quantities of viral biomarkers^10,11^ are essential.^12,13^ While there have been reports of highly sensitive sensors^14–17^ developed for JEV antigen (Ag) detection, most of them are electrochemical-based and expensive as well as difficult to fabricate, require skilled handling, and most importantly, are not portable point-of-care (PoC) devices.^18–24^ To overcome these limitations of existing sensors, Ag targeting immunochromatographic lateral flow assays (LFAs) are being developed for rapid bedside detection of various infectious diseases.^25,26^ LFAs comprise prefabricated strips of a carrier probe containing dry reagents that are activated by addition of a fluid sample,^27^ and the absence/presence of the target analyte is indicated by the appearance of coloured lines on the membrane.^28^ These colorimetric assays are inexpensive, and easy to fabricate, rapid, portable, and user-friendly.^29,30^ Since LFAs are portable, they can be used for on-site detection,^31^ especially for mass-screening of JEV in human as well as pig serum, which is most prevalent in rural areas.^32,33^ Based on existing literature, the virus is known to seroconvert in swine 2–4 weeks before human infection and hence pig serum may be used for the screening of the JEV NS1 protein for early detection, surveillance, and prevention of an outbreak in humans.^34^ Based on this, a LFA has been developed for the sero-diagnosis of JEV antibodies (Abs) in swine,^35^ but no such PoC has been developed for the JEV secretory NS1 biomarker in serum, which is more advantageous since the Ag is found in the secretory system from the first day of infection (CDC Report (https://www.cdc.gov/dengue/healthcare-providers/testing/antigen-detection.html)) enabling detection at an early stage before seroconversion, whereas Abs are produced after the fourth or fifth day of infection.^36^

In this report, we have developed a colorimetric sandwich-based LFA^37–39^ using gold nanoparticles (AuNPs) labelled with the JEV NS1 Ab^40^ for rapid, sensitive, and specific JEV NS1 Ag detection in serum samples. We have made use of the surface plasmon resonance (SPR) properties that contribute to AuNPs' characteristic wine-red colour as the chromatic indicator, while the antibody acts as a specific immunoprobe. Besides optical properties (which enhance the signal intensity), AuNPs are also easy to synthesise as well as bioconjugate, and increase the surface area for maximum Ab *i.e.* bioreceptor attachment.^41^ JEV NS1 polyclonal Abs were generated against an in-house expressed JEV NS1 recombinant protein,^14^ while the AuNPs were synthesised by the heat-reflux citrate reduction method.^42^ Compared to monoclonal Abs as bioreceptors, polyclonal Abs are more cost effective and require less generation time, recognise multiple epitopes giving them an overall higher binding affinity to the target antigen, which in turn increases sensitivity, are able to detect minute quantities of the target antigen in the sample, and have a higher chance of showing affinity towards mutating antigens.^43^ The AuNPs–Ab immuno-chromatic probe solution was used for the fabrication of a multi-membrane layered LFA strip. Different fabrication parameters of the developed immunosensor such as AuNPs–Ab conjugate concentration, test line JEV NS1 1° Ab concentration, NC membrane pore size, and blocking solution composition were standardised, and tested using the JEV NS1 Ag in the range 1 pg ml^−1^ to 1 μg ml^−1^ by spiking JEV negative serum. The naked eye visual limit of detection (LOD) determined from the strips was 10 pg ml^−1^, which corresponded to the LOD determined from the graphical data obtained from both ImageJ computer software and the Colorimeter smartphone application, which can measure the minimum concentration of circulating NS1 protein required to cause infection ranging from 7–284 ng ml^−1^ as reported in other flaviviral clinical samples.^44^ Detection in serum samples over cerebrospinal fluid is preferred as it is less invasive and serum does not contain the whole virus, which removes the need for a Biosafety Laboratory 3 (BSL-3) facility due to the highly infectious nature of JEV, making it less hazardous for the diagnostician. The fabricated LFA was able to detect JEV NS1 Ags in spiked serum samples and showed minimal non-specific binding against other cross-reactive flaviviral NS1 Ags with rapid results within 10 min and storage stability for 1 month at 4 °C. Finally, the fabricated LFA strips were used to successfully detect the NS1 protein in clinically infected JEV pig serum samples. Hence, the designed optical immunosensor may be developed into a novel kit for mass screening of JEV for on-site PoC detection, especially in rural areas and clinics and can further also be integrated with artificial intelligence and the internet of things to create prototypes of 5^th^ generation sustainable intelligent sensing systems, including wearable sensors.^45–48^

## 2. Experimental

### 2.1 Chemicals

Sodium citrate tribasic dehydrate (C_6_H_5_Na_3_O_7_·2H_2_O), sodium chloride (NaCl), sodium hydroxide (NaOH), potassium chloride (KCl), bovine serum albumin (BSA), potassium dihydrogen orthophosphate (KH_2_PO_4_), uranyl acetate, and tris base (C_4_H_11_NO_3_) were obtained from Sisco Research Laboratories (SRL, India). Sodium dihydrogen phosphate-1-hydrate (Na_2_HPO_4_·H_2_O) and sodium tetraborate (Na_2_B_4_O_7_) were purchased from Merck (Mumbai, India). Gold(iii) chloride (Au_2_Cl_6_), boric acid (H_3_BO_3_), and skimmed milk powder were procured from Sigma Aldrich (India). Hydrochloric acid (HCl) and ethanol were procured from Fisher Scientific (India), while anti-rabbit immunoglobulin G (IgG) antibodies were purchased from G-Biosciences (Noida, India). Dengue-2 Virus, West Nile Virus, and Yellow Fever Virus NS1 proteins were acquired from The Native Antigen Company (Oxfordshire, UK). The Japanese Encephalitis Virus NS1 Ag and Ab were developed in the lab as explained in earlier work.^14^ JEV positive and negative pig serum real samples were a kind gift from the ICAR-Indian Veterinary Research Institute (IVRI) (Uttar Pradesh, India). All high analytical grade reagents were used and all solutions were prepared in double distilled water unless mentioned otherwise.

### 2.2 Apparatus

Elemental analysis of the synthesised AuNPs was conducted *via* X-ray Photoelectron Spectroscopy (XPS) on a Kratos Analytical AXIS Supra+ (Manchester, UK). UV-Vis spectra were obtained using a Systonic S-924 Single-Beam UV-Vis Spectrophotometer (Delhi, India). The zeta potential and average hydrodynamic diameter during AuNP labelling of the Ab were acquired on an Anton Paar Litesizer 500 Dynamic Light Scattering (DLS) instrument functioning at 532 nm/50 mW laser excitation (green wavelength source) (Graz, Austria). The size, morphology, and monodispersity of nanoparticles before and after conjugation were visualised on a JEOL JEM-2100 Transmission Electron Microscope (Tokyo, Japan) functioning at 200 kV with samples coated on Ted Pella Inc. Formvar copper grids (Redding, California, USA). The topological characteristics of the AuNP surface were studied *via* an Oxford Instruments-MFP-3D Origin Atomic Force Microscope (Abingdon, UK) using AR 16.25 software. The test and control lines were dispensed onto a nitrocellulose (NC) membrane for LFA fabrication using an Easy Printer Model LPM-02 supplied by mdi Membrane Technologies, Advanced Microdevices (Haryana, India) along with the various membranes and plastic cassette cases required for the development of the LFA. 2.5 mm LFA strips were uniformly cut using a programmable RTSC-750 rapid test strip cutting machine purchased from Global Scientific Equipment (Haryana, India). A One Plus 7 smartphone (GM1901) with a 48+5 MP dual rear camera (Shenzhen, China) was used to capture the individual LFA strip images under artificial white light. ImageJ software v.1.52 (ref. 49) was employed to analyse the mean light intensity of colour development of bands during experiments. The Colorimeter Smartphone Application v.1.6.6.2 (ref. 50 and 51) (Research Lab Tools, Sao Poulo, Brazil) was used to obtain the red, blue and green pixel values of each LFA strip test line. All experiments were repeated thrice and conducted at 25 °C room temperature (RT) unless mentioned otherwise.

### 2.3 Synthesis, bioconjugation, and characterisation of the AuNPs–Ab immunoprobe

Monodispersed AuNPs were synthesised using a modified heat-reflux based reduction process using citrate.^52,53^ 10% gold chloride was mixed with distilled water in an Erlenmeyer flask and brought to the boiling point. 1% w/v sodium citrate was quickly added to the boiling mixture and allowed to boil until the colour of the solution gradually changed from light yellow to the characteristic wine-red colour of colloidal AuNPs. The solution was then allowed to cool and stored at 4 °C for further experimentation. Elemental analysis of the synthesised gold nanoparticles was carried out using X-ray Photoelectron Spectroscopy (XPS). The JEV NS1 Ab was generated in New Zealand White rabbits against an in-house expressed JEV NS1 recombinant protein, purified using Protein-A Sepharose columns, put on dialysis in Phosphate Buffer Saline (PBS) (pH 7.4) and characterised for its binding affinity against different flaviviral NS1 Ags *via* ELISA (LOD of 0.94 μg ml^−1^ in buffer and 1.16 μg ml^−1^ in serum) as explained in our previous research.^14^

For AuNPs–JEV NS1 Ab labelling, 36 μg of JEV NS1 Ab was dropwise introduced into 3 ml AuNP colloidal suspension in borate buffer, mixed and incubated at RT. Bovine serum albumin (BSA) in borate buffer was added to the solution and mixed followed by incubation at RT. The mixture was then centrifuged and the supernatant was carefully discarded to remove excess unbound Abs. The pellet was resuspended in borate buffer, mixed and again centrifuged as a washing step to eradicate the remaining unbound Abs. The resultant AuNPs–Ab immuno-chromatic probe was finally resuspended in borate buffer and stored at 4 °C for further experimentation.

Different techniques were performed to characterise the labelling of the JEV NS1 Ab with AuNPs such as UV-Vis spectroscopy ranging from 200–800 nm for bare AuNPs and the bioconjugate to detect the shift in the wavelength of light absorbed. The zeta potential and average hydrodynamic diameter were acquired using Dynamic Light Scattering (DLS) performed at 532 nm/50 mW green laser excitation to observe an increase in the size and decrease in monodispersity/stability upon AuNPs–Ab conjugation. The average hydrodynamic diameter was calculated using the Stokes–Einstein equation with the continuous phase being considered as aqueous, where the viscosity of water was 0.911–0.852 mPa s^−1^ and the AuNP diffusion coefficient was 6.89 × 10^−9^ to 5.30 × 10^−8^ cm^2^ s^−1^.^54^ The samples were drop cast onto carbon coated Formvar copper grids and negatively stained with uranyl acetate, to study the size, morphology, distribution, and conjugation of bare AuNPs and AuNPs–Ab visualised *via* Transmission Electron Microscopy (TEM). Similarly, the samples were deposited onto silicon wafers before and after conjugation to observe the changes in the topological characteristics by studying the change in surface signal intensity *via* Atomic Force Microscopy (AFM).

### 2.4 Fabrication and optimisation of multi-layered strip-based lateral flow immunoassay

A lateral flow immunoassay was developed using the following membrane layers: nitrocellulose (NC) membrane pad, glass fibre conjugate pad, cellulose sample pad and cellulose absorbent pad. All RT pre-treatment and fabrication steps were performed in a clean-air flow chamber. The sample and conjugate pads were pre-treated before assembly. The pre-treatment of the sample and conjugate pads is necessary for membrane equilibration, anti-fouling of the membrane pores, and uniform easy flow of the sample/conjugate across the membranes. For AuNPs–Ab conjugation, the amount of Ab used was optimised between 4.5 μg, 9 μg, 18 μg, and 36 μg in 3 ml AuNPs, loaded onto the pre-treated conjugate pad and allowed to dry. The JEV NS1 Ab and anti-rabbit IgG 2° Ab were coated as the test and control lines respectively on the NC membrane using an easy printer. Different concentrations of JEV NS1 Ab on the test line (0.125, 0.25, 0.5, 1, and 2 mg ml^−1^) as well as different pore sizes of the NC membrane (8, 10, 12, and 15 μ) were optimised for maximum signal intensity at the test line. Once dry, the remaining surface of the NC membrane, where no Ab was attached, was blocked using BSA in different buffers such as Tris, phosphate, PBS and borate buffer to avoid non-specific binding during testing. The optimised blocking buffer was added to the NC membrane and dried at 37 °C. Post incubation, the conjugate pad plus absorbent pad was placed at two ends of the NC membrane ensuring that both the pads overlap the NC by 1–2 mm. The sample pad was pasted over the conjugate pad on the laminate. Once assembled, uniform 2.5 mm thick LFA strips were cut using a rapid test strip cutting machine and placed in individual plastic cassettes for testing.

### 2.5 Analytical performance of the fabricated lateral flow immunoassay

Variable JEV NS1 Ag concentrations were prepared in JEV negative serum at 10-fold dilutions in the linear range 1 pg ml^−1^ to 1 μg ml^−1^ and 100 μl was added to the sample wells of individual LFA cassettes. The spiked serum was mixed with chase buffer in a ratio of 1 : 10 for the ease of flow across the LFA strip. The development of colour in the test and control lines was observed after 10 min and images were captured under white light. The mean intensity of the test line was measured using ImageJ software v.1.52 and plotted against JEV NS1 Ag concentration in pg ml^−1^. Similarly, the red, blue, and green pixel values were obtained from the Colorimeter Smartphone Application (app) v.1.6.6.2 and plotted against JEV NS1 Ag concentration in pg ml^−1^. The limit of detection (LOD) was determined from the intensity of test line colour development by the naked eye and corroborated with the LOD results obtained from ImageJ software and Colorimeter app graphs. Furthermore, cross reactivity and matrix effect studies were carried out by testing the LFA against 1 μg ml^−1^ JEV, Yellow Fever Virus (YFV), West Nile Virus (WNV), and Dengue Virus (DENV) NS1 Ag spiked in JEV negative serum and the results were compared to those of negative control serum. Finally, the fabricated LFA strip was used for JEV NS1 Ag detection in 20 clinically infected JEV pig serum samples. The assembled and coated LFA strips were also stored at RT as well as 4 °C to analyse the stability for a month at weekly intervals.

## 3. Results and discussion

### 3.1 Working principle of the fabricated lateral flow immunoassay

**Fig. 1:**
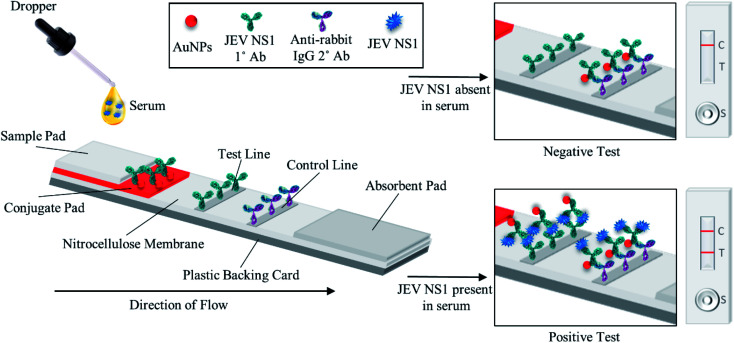
The fabricated LFA strip consists of a plastic backing card with overlapping membranes, a porous nitrocellulose membrane coated with the JEV NS1 Ab and anti-rabbit IgG 2° Ab on test and control lines respectively, a conjugate pad with the AuNPs–JEV NS1 Ab conjugate, and a pre-treated sample pad at one end and an absorbent pad at the other end. In the case of negative samples, only the control line develops, whereas in the case of positive samples, both test and control lines develop.

As shown in the schematic (Fig. 1), the developed sandwich based LFA is based on the capillary movement of the sample in the liquid form, which contains the target analyte (JEV NS1 Ag) across various overlapping membrane layers precoated with molecules that may interact with the target biomarker. The LFA strip is composed of different overlapping membranes stabilised on a hard plastic backing card base. The sample is added to the pre-treated sample pad at one end of the assembled LFA, making it suitable for interaction with the detector immuno-chromatic probe (AuNPs–Ab) on the conjugate pad. The sample pad is made up of cellulose which is inexpensive, helps absorb the entire sample and increases its viscosity due to its thickness, promotes even flow of the sample along the membrane, and enables efficient binding of the NS1 target protein in the sample with the capture immuno-chromatic probe (AuNPs–Ab) on the conjugate membrane. From the sample pad, capillary migration of the liquid sample occurs across the conjugate release membrane, where the immunoprobe JEV NS1 Abs, specific to the target JEV NS1 Ag, are present in the conjugated state with chromatic red colloidal gold nanoparticles. The glass fibre material of conjugate pads can hold up large volumes, shows low non-specific binding, and is able to deliver the detector particles onto the membrane in a consistent volume of sample on every test strip. The JEV NS1 Ag captured by the specific AuNPs–JEV NS1 Ab immuno-chromatic probe forms a complex that migrates along the NC membrane, which is porous with good capillary action for ease of flow of the sample and conjugate, into the zone of detection. The JEV NS1 Ab and anti-rabbit IgG 2° Ab were coated as test plus control lines respectively in the test zone of the porous NC membrane. The development of red coloured bands on the test and control lines with varying intensities may be analysed visually by the naked eye. To maintain the flow of liquid *via* capillary action across the LFA, a wicking absorbent pad made of thick inexpensive cellulose is attached on the opposite end from the sample pad, which soaks up the chase buffer and prevents the backflow of reagents.

In the case of negative samples, no JEV NS1 Ag attaches to the AuNPs–Ab conjugate and hence only the immuno-chromatic AuNPs–Ab probe flows through the NC membrane and gets captured only at the control line by the anti-rabbit IgG 2° Ab resulting in the development of only control line. However, in the case of positive samples, the JEV NS1 Ag present attaches to the immuno-chromatic AuNPs–Ab probe and the entire complex gets captured by the JEV NS1 1° Ab at the test line while the excess AuNPs–Ab conjugate gets captured by the anti-rabbit IgG 2° Ab at the control line, resulting in the development of the test as well as the control line.

### 3.2 Characterisation of the synthesised AuNPs and its conjugation with the JEV NS1 Ab

**Fig. 2:**
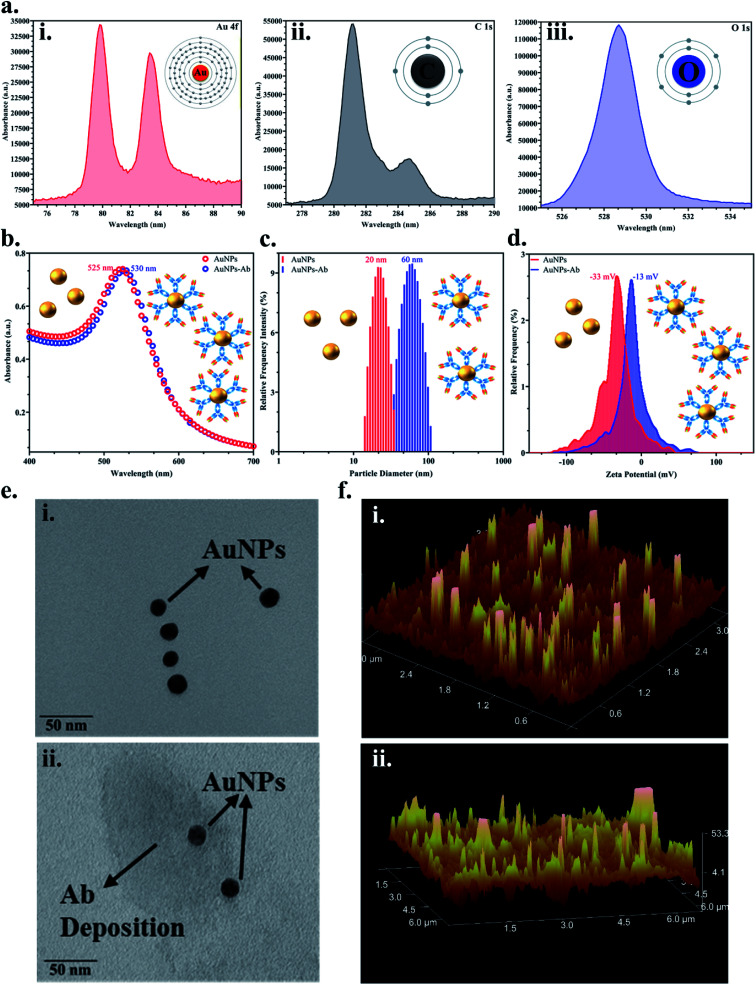
Characterisation of the synthesised AuNPs before and after bioconjugation with the JEV NS1 Ab: (a) XPS data representing the characteristic elemental peaks of (i) Au 4f, (ii) C 1s, and (iii) O 1s; (b) UV-Vis spectra showing a red shift in the wavelength from 525 to 530 nm upon bioconjugation of the Ab on AuNPs; (c) increase in the average hydrodynamic diameter observed in DLS by 40 nm after conjugation; (d) zeta potential graph showing shift in surface charge from negative towards positive before and after Ab conjugation; (e) TEM images showing (i) monodispersed 20 ± 5 nm AuNPs and (ii) Ab protein deposition around the AuNPs after conjugation; (f) AFM surface morphology studies of (i) bare AuNPs and (ii) the AuNPs–Ab conjugate.

Colloidal monodisperse AuNPs were synthesised and the bioconjugation with the JEV NS1 Ab was confirmed *via* various methods. Firstly, the elemental analysis of the colloidal AuNPs *via* XPS showed peaks of Au 4f (corresponding to the formation of gold from Au_2_Cl_6_) (Fig. 2a(i)), C 1s (corresponding to citrate used in the synthesis process) (Fig. 2a(ii)) and O 1s (corresponding to citrate used in the synthesis process) (Fig. 2a(iii)), which confirmed the characteristic elemental fingerprint pattern of citrate heat-reflux synthesised AuNPs. The UV-Vis graph (Fig. 2b) displayed the distinctive peak of bare AuNPs at 525 nm, while the AuNPs–Ab conjugate showed a red shift of 5 nm *i.e.*, at 530 nm due to the labelling of AuNPs with the Ab. This increase in size was corroborated by analysing the average hydrodynamic data obtained from DLS (Fig. 2c), where the bare AuNPs were of 20 nm average size, whereas after conjugation, the average size of the aggregated AuNPs–Ab increased to 60 nm. We have used 20 nm AuNPs based on previous literature, 20–30 nm AuNPs have the ideal size for LFA development due to the ease of flow and conjugation and they do not aggregate easily.^31,55^ In Fig. 2d, the zeta potential shift was observed from −33 mV to −13 mV, which is a shift from negative to positive charge. Since AuNPs were synthesised using the citrate heat-reflux method, they showed a higher negative surface charge as well as more repulsion and were stable; however, the addition of the Ab upon conjugation reduced stability and resulted in aggregation, which shifted the surface charge towards positive.

In Fig. 2e(i), the TEM image confirmed that the synthesised AuNPs were monodispersed and approximately 20 ± 5 nm in size and upon conjugation, a proteinaceous Ab deposition was observed around AuNPs (Fig. 2e(ii)). AFM images showed increase in signal intensity while analysing the topology and surface structure of bare AuNPs (Fig. 2f(i)), and the AuNPs–Ab bioconjugate (Fig. 2f(ii)) due to increase in the surface roughness and height, when the Ab was immobilised on the AuNP surface, which further confirmed the bioconjugation.

### 3.3 Optimisation of multi-layered strip-based lateral flow immunoassay

**Fig. 3:**
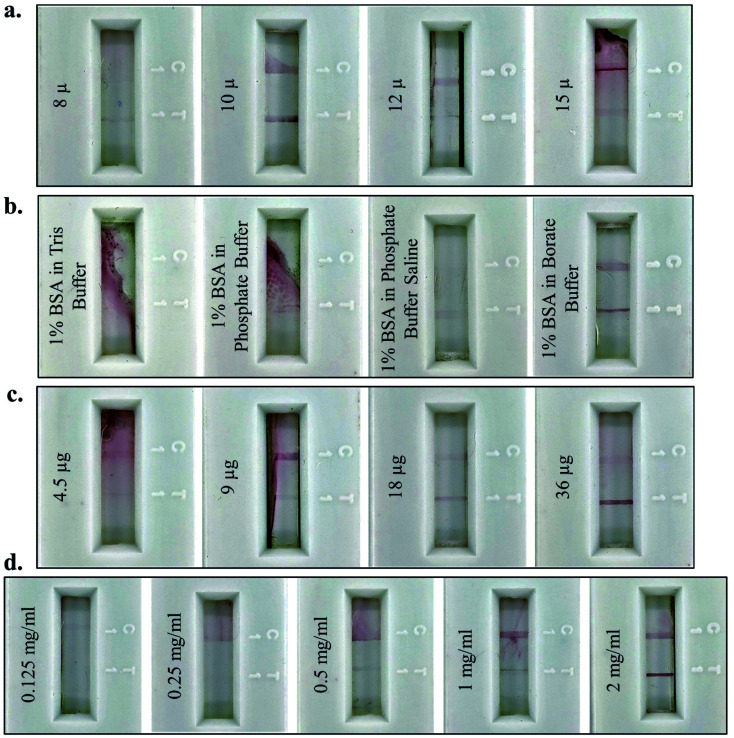
Optimization of LFA fabrication: (a) optimisation of various pore sizes of the NC membrane (8 μ, 10 μ, 12 μ, and 15 μ) for the ease of flow; (b) selection of ideal blocking buffer *i.e.* BSA in Tris buffer, phosphate buffer, phosphate buffer saline or borate buffer, to prevent non-specific binding of the NC membrane; (c) variable concentration of the JEV NS1 Ab for conjugation with AuNPs (4.5 μg, 9 μg, 18 μg and 36 μg); (d) different concentrations of the JEV NS1 Ab (0.125 mg ml^−1^, 0.25 mg ml^−1^, 0.5 mg ml^−1^, 1 mg ml^−1^ and 2 mg ml^−1^) for test line coating on the NC membrane.

For optimum results, different parameters for the fabrication of the lateral flow assay strip were standardised. NC membranes of varying pore sizes (8 μ, 10 μ, 12 μ, and 15 μ) were used to fabricate the assay strip and the maximum intensity of the test and control lines was observed on 10 μ pore size NC (Fig. 3a). This confirmed that 10 μ pores were of optimum size for easy flow across the NC membrane for JEV NS1 Ag present in serum (1 : 10 dilution of serum in chase buffer), the AuNPs–JEV NS1 Ab bioconjugate on the conjugate pad, and the complex formed between the two. Hence, 10 μ NC was selected for further fabrication of the LFA strips. After coating of the Ab on the test and control lines, blocking was carried out using different buffer combinations (Tris, phosphate, PBS and borate buffer) with BSA to block the remaining surface of the NC membrane and prevent non-specific binding. Proper flow of the sample with the conjugate and distinct development of test and control lines without any background staining were observed only by blocking with BSA in borate buffer (Fig. 3b), which was further used for blocking of the Ab coated test strips. The higher pH of borate buffer as compared to the other three buffers prevents the aggregation of the AuNPs–Ab conjugate on the NC membrane as it flows along with the sample and does not affect the functioning of the LFA.

Furthermore, the antibody conjugation and coating parameters were standardised for maximum colour development of the test line. Variable concentration of Ab (4.5 μg, 9 μg, 18 μg and 36 μg) were used for conjugation with the colloidal AuNPs (Fig. 3c) to achieve maximum test line signal intensity, with no background staining, which was observed at 36 μg of Ab and further selected as the optimal conjugation. Finally, the concentration of JEV NS1 Ab (0.125 mg ml^−1^, 0.25 mg ml^−1^, 0.5 mg ml^−1^, 1 mg ml^−1^ and 2 mg ml^−1^) required for coating on the test line was standardised with maximum binding efficiency at 2 mg ml^−1^ as observed in Fig. 3d, which was selected as the optimum coating concentration for LFA fabrication.

### 3.4 Analytical performance of the fabricated lateral flow immunoassay

**Fig. 4:**
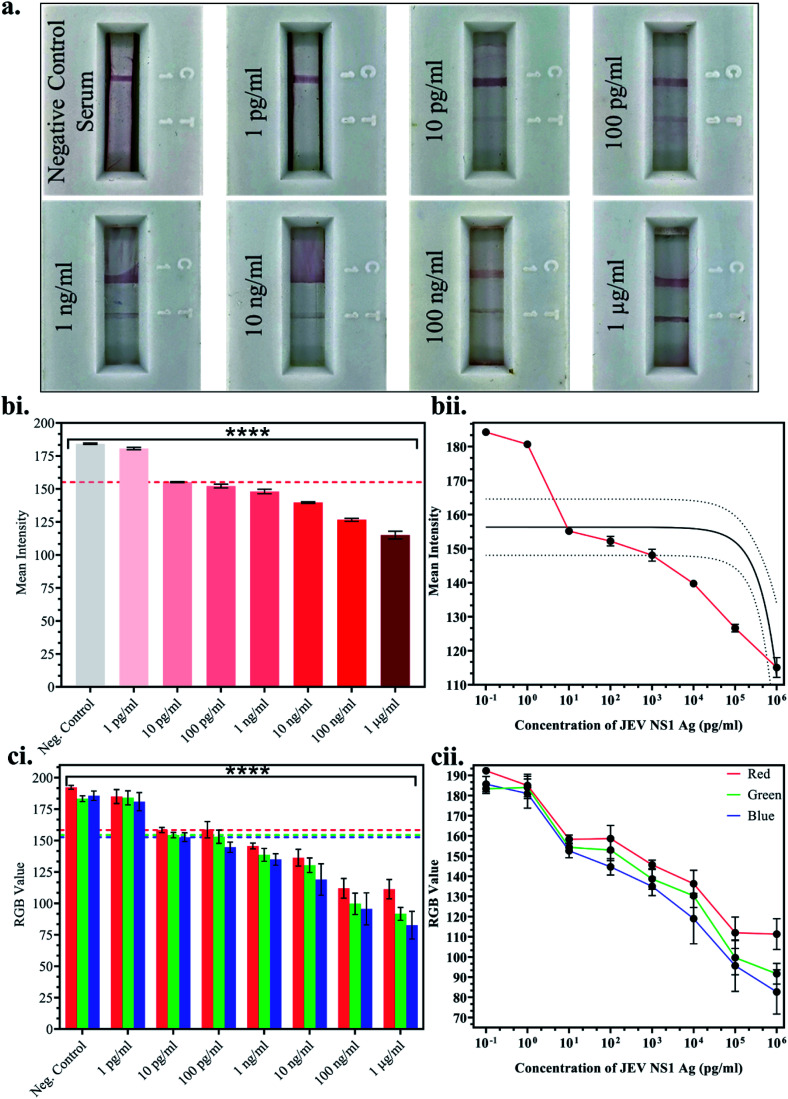
Analytical testing of the fabricated LFA strips: (a) images of individual LFA strips for the detection of 10-fold JEV NS1 Ag dilutions spiked in JEV negative serum ranging from 1 pg ml^−1^ to 1 μg ml^−1^; (b) ImageJ computer software test line mean intensities compared against Ag concentration and plotted as (i) a bar graph and (ii) a linear calibration graph; (c) smartphone colorimeter application test line RGB values compared against Ag concentration and plotted as (i) a bar graph and (ii) a linear calibration graph.

Once the above parameters were successfully standardised, multiple LFA strips were fabricated for the detection of a range of JEV NS1 Ag concentrations spiked in JEV negative serum. 10-fold dilutions ranging from 1 pg ml^−1^ to 1 μg ml^−1^ of JEV NS1 Ag were tested on the LFA strips and the test line colour intensity was observed to decrease by the naked eye from higher to lower protein concentration (Fig. 4a). Since, clear test line as compared to the negative control serum strip was observed at 10 pg ml^−1^ and beyond, 10 pg ml^−1^ was selected as the naked eye Limit of Detection (LOD). Furthermore, the captured images of the developed LFA strips were uploaded onto ImageJ computer software and the white colour mean intensity of the test line was noted and a bar graph was plotted (Fig. 4b(i)) as well as a linear calibration graph (Fig. 4b(ii)). Decrease in the white colour mean intensity was observed with increase in Ag concentration, as the red colour increased. From the graphs, it was clear that the intensity reading began to significantly decrease beyond 10 pg ml^−1^, which was determined as the LOD *via* ImageJ software with *p* < 0.0001. Similarly, a smartphone based colorimeter application for on-site application was used to detect the Red-Green-Blue (RGB) pixel value of the test line in the captured images of the LFA strips, and a bar graph was plotted (Fig. 4c(i)) as well as a linear calibration graph (Fig. 4c(ii)). The obtained results could also be recorded *via* live imaging options on the app. In a colour spectrum, RGB values tend towards a minimum 0 value for black and a maximum 255 value for white.

Hence, it was observed that at the lowest Ag concentration when the test line colour intensity was lighter red (tending towards white), the RGB values were higher, whereas at the highest Ag concentration when the test line colour intensity was darker red (tending towards black), the RGB values were lower. From the graphs, it was found that the RGB values began to significantly decrease beyond 10 pg ml^−1^, which was determined as the LOD *via* the smartphone colorimeter application with *p* < 0.0001. This proved that the portable real-time on-site mobile application was as efficient as the standardised computer ImageJ software without any loss in sensitivity or effect on results.

### 3.5 Cross-reactivity studies of the fabricated LFA against other flaviviral NS1 Ag

**Fig. 5:**
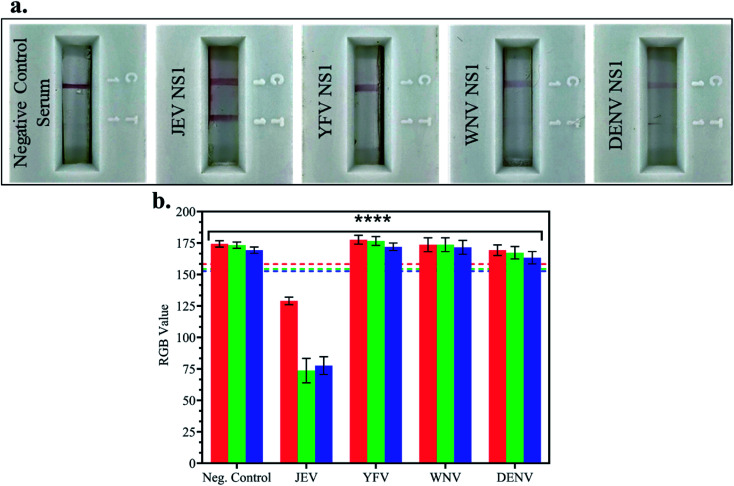
Specificity studies of the fabricated LFA against other cross-reactive flaviviral NS1 Ags: (a) LFA strip images of testing 1 μg ml^−1^ NS1 Ag of different *Flaviviruses* spiked in JEV negative serum; (b) bar graph of the RGB values of LFA cross-reactive test strips using the smartphone colorimeter application compared to the RGB lines of the LOD (10 pg ml^−1^) as the positive control.

In JEV negative serum, 1 μg ml^−1^ NS1 Ags of different *Flaviviruses* – Japanese Encephalitis Virus, Yellow Fever Virus (YFV), West Nile Virus (WNV) and Dengue Virus (DENV) – were spiked and tested on LFA strips, and the RGB values of the test line were compared with the LOD (10 pg ml^−1^) RGB values from the previous experiment. In Fig. 5a LFA strip images, red test line development was observed when JEV NS1 Ag spiked in JEV negative serum was added to the LFA strip but no test line was developed in the case of negative control serum, YFV NS1, WNV NS1, and DENV NS1 Ag. This confirmed that the fabricated LFA was specific for JEV NS1 Ag detection and did not show non-specific binding with similar cross-reactive flaviviral species NS1 Ag.

Furthermore, there was no development of the test line due to other components present in serum as seen in the negative control, negating the matrix effect which may have resulted in false positive outcomes. The RGB values using the smartphone colorimeter application were recorded for cross-reactive testing LFA strip images, and the bar graphs were plotted for each NS1 sample in spiked JEV negative serum (Fig. 5b) and compared with the LOD (10 pg ml^−1^) RGB value with *p* < 0.0001. It was observed that the samples containing JEV NS1 Ag showed RGB values lower than the LOD (tending towards black), whereas the samples containing NS1 Ag of cross-reactive *Flavivirus* showed RGB values higher than the LOD (tending towards white) similar to the negative control serum sample. Hence, the naked eye analysis of LFA cross-reactivity was confirmed by the RGB values obtained from the handheld smartphone colorimeter application.

### 3.6 JEV infected clinical serum sample analysis using the fabricated LFA

**Fig. 6:**
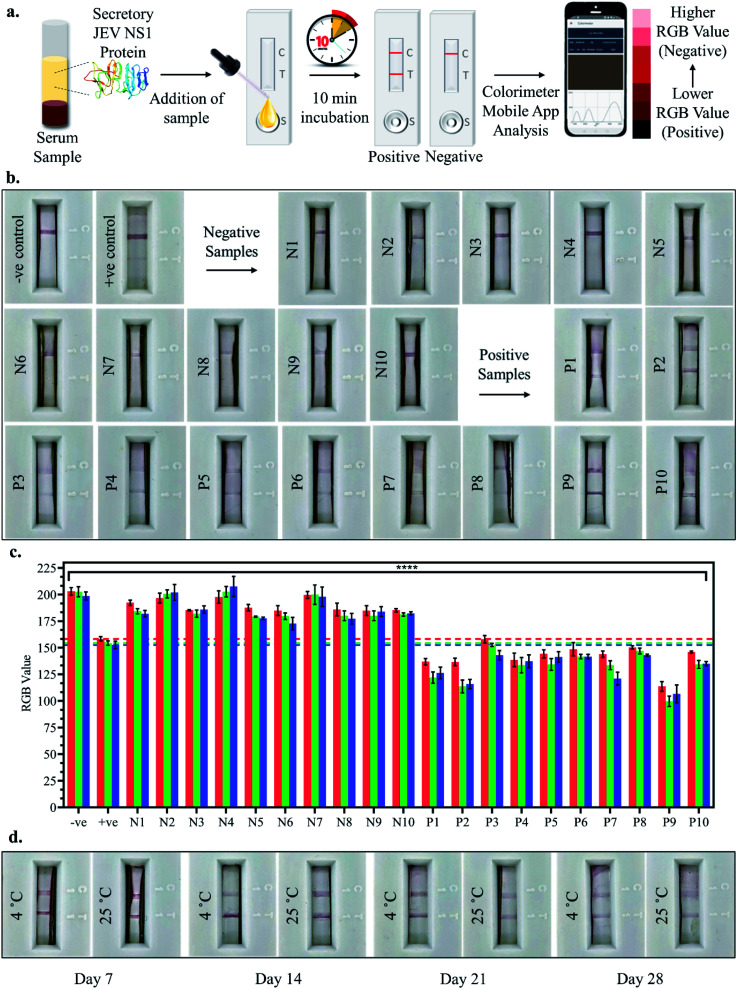
JEV NS1 Ag detection in clinical serum samples: (a) schematic representation of the diagnostic workflow on the fabricated LFA and smartphone colorimeter application analysis; (b) JEV negative and positive serum sample LFA images; (c) bar graph of the RGB values of 20 clinical samples using the smartphone colorimeter application; (d) storage stability of the fabricated strips at 4 °C and RT for one month.

10 positive and 10 negative JEV infected clinical pig serum samples were tested by adding a 100 μl sample (serum sample diluted 10-fold in chase buffer) to the LFA strips and the test line development was observed after 10 min incubation, both by the naked eye and smartphone colorimeter app RGB value analysis as shown in Fig. 6a. Upon visual naked eye detection, no test line development was observed in the case of JEV negative serum samples (N1–N10), which corresponded to the negative control serum sample as observed in Fig. 6b. Similarly, in the case of all JEV positive serum samples (P1–P10), clear development of the red test line was visualised by the naked eye (Fig. 6b). The bar graph plot of the RGB values of the negative sample LFA strip test lines (N1–N10) revealed that the RGB values were all greater than those of the positive control serum *i.e.*, LOD (10 pg ml^−1^) with *p* < 0.0001, similar to the negative control serum, tending towards white (Fig. 6c). However, from the bar graph of the RGB values of the positive sample LFA strip test lines (P1–P10), it was found that the RGB values were all lower than the positive control serum *i.e.*, LOD (10 pg ml^−1^) with *p* < 0.0001, tending towards black (Fig. 6c). Hence, the developed LFA strip was able to successfully detect all the clinically positive JEV serum samples with 100% sensitivity using both the naked eye and smartphone application-based detection, making it an ideal PoC diagnostic tool. Finally, coated and assembled LFA strips were stored for a month at 4 °C and RT and tested at weekly intervals, and the test lines for JEV NS1 positive samples were found to develop even after a month of storage at both 4 °C and RT (Fig. 6d). However, the intensity of the test line developed was lighter in the case of RT and after the 4^th^ week, both the intensity of the test line as well as flow rate across the test strip diminished substantially and hence, the fabricated LFA may be assembled and stored at 4 °C for a period of 1 month in a dry place without any decrease in detection capacity, which enables transportation in a dry environment to testing sites.

## 4. Conclusions

This study reports the fabrication of a lateral flow colorimetric assay using a AuNP labelled JEV NS1 Ab immuno-chromatic probe for the rapid, sensitive, and specific detection of JEV NS1 Ag in clinical serum samples. The visual limit of detection of this portable, cost effective, and disposable immunochromatographic strip was found to be 10 pg ml^−1^ for JEV NS1 Ag spiked in JEV negative serum, in the range 1 pg ml^−1^ to 1 μg ml^−1^ which was further corroborated *via* ImageJ computer software and the on-site smartphone colorimeter application. Hence, the developed LFA can detect the minimum concentration of circulating NS1 required to cause an infection ranging between 7 and 284 ng ml^−1^ as reported in other flaviviral clinical samples. Furthermore, it was also able to successfully detect JEV NS1 Ag in JEV infected clinical pig serum samples within a rapid response time of 10 min using both naked eye detection or live imaging RGB value detection *via* the smartphone colorimeter application. The proposed immunosensor showed negligible non-specific binding with other cross-reactive flaviviral NS1 Ags (WNV, YFV and DENV) in spiked JEV negative serum. Moreover, the fabricated LFA strips could be stored for up to a month at 4 °C in a dry environment after coating and assembly before testing. Hence, this easy to fabricate, sensitive, specific, cost-effective, disposable and user-friendly LFA may potentially be developed into a novel PoC diagnostic kit for on-field rapid mass screening, especially in rural areas where there is a dearth of laboratory set-ups and skilled personnel.

## Ethics approval

All biosafety and animal related experiments were carried out at the National Institute of Animal Biotechnology (DBT-NIAB), Hyderabad after due approval from the Institutional Biosafety Committee (IBSC) with approval number #IBSC/2019/NIAB/Sonu002 and Institutional Animal Ethics Committee (IAEC) with approval number IAEC/2019/NIAB/26/SG.

## Author contributions

Sonu Gandhi: conceptualization, supervision, visualization, writing – review, funding acquisition, and project administration. G. Taru Sharma: discussion, funding acquisition, and project administration. Himani Dhanze and Ravi Kumar Gandham: clinical sample acquisition. Akanksha Roberts: writing – original draft and editing, experimentation, investigation, analysis, and data presentation. Drishya Prakashan: writing – original draft, experimentation, investigation, and analysis.

## Conflicts of interest

The authors have no conflict of interest to disclose.

## Supplementary Material
